# Common Nevus and Skin Cutaneous Melanoma: Prognostic Genes Identified by Gene Co-Expression Network Analysis

**DOI:** 10.3390/genes10100747

**Published:** 2019-09-25

**Authors:** Lingge Yang, Yu Xu, Yan Yan, Peng Luo, Shiqi Chen, Biqiang Zheng, Wangjun Yan, Yong Chen, Chunmeng Wang

**Affiliations:** 1 Department of Musculoskeletal Oncology, Fudan University Shanghai Cancer Center, Shanghai 200032, Chinaxuyudaniel@gmail.com (Y.X.); michelle_yan@fudan.edu.cn (Y.Y.); Zhengbqk@hotmail.com (B.Z.); yanwj@fudan.edu.cn (W.Y.); chenyong@fudan.edu.cn (Y.C.); 2 Department of Oncology, Shanghai Medical College, Fudan University, Shanghai 200032, China

**Keywords:** skin cutaneous melanoma, common nevus, GSE98394, weighted gene co-expression network analysis (WGCNA), prognostic genes

## Abstract

Skin cutaneous melanoma (SCM) is a common malignant tumor of the skin and its pathogenesis still needs to be studied. In this work, we constructed a co-expression network and screened for hub genes by weighted gene co-expression network analysis (WGCNA) using the GSE98394 dataset. The relationship between the mRNA expression of hub genes and the prognosis of patients with melanoma was validated by Gene Expression Profiling Interactive Analysis (GEPIA) database. Furthermore, immunohistochemistry in the Human Protein Atlas was used to validate hub genes and grayscale analysis was performed using ImageJ software. It was found that the yellow module was most significantly associated with the difference between common nevus and SCM, and 13 genes whose expression correlation >0.9 were candidate hub genes. The expression of three genes (*STK26, KCNT2, CASP12*) was correlated with the prognosis of SCM. STK26 (*P* = 0.0024) and KCNT2 (*P* < 0.0001) were significantly different between normal skin and SCM. These three hub genes have potential value as predictors for accurate diagnosis and prognosis of SCM in the future.

## 1. Introduction

Skin cutaneous melanoma (SCM) is a highly aggressive malignant tumor that originates from neural crest melanocytes and is triggered by hyperplasia of abnormal melanocytes. According to data released by GLOBOCAN online database (gco.iarc.fr), in 2018, there were 287,723 new cases (1.6% of the total cases) of SCM in the world, and 60,712 of these patients (0.6% of total cancer deaths) died [1]. SCM is characterized by a high metastasis rate, high mortality, and strong drug resistance. The main causes of death include extensive metastasis of the lung, liver, bone, or brain [2]. Patients with SCM potentially lose 20.4 years of their lifespan, which is significantly higher than the 16.6 years for all other malignant tumors [3]. Therefore, SCM has become one of the malignant tumors that seriously threaten human health. For patients with stage I and II melanoma, the 10-year melanoma-specific survival ranged from 98% to 75% [4], so early diagnosis and treatment of SCM are necessary. The phenotypic predispositions in the risk factors of SCM included atypical mole or dysplastic nevus pattern [5] and increased mole count (particularly large nevi) [6]. However, the transformation mechanism of nevus into SCM is still unclear, and further research is urgently needed.

The weighted gene co-expression network analysis (WGCNA) is a systematic biological method to describe the pattern of gene association between different samples. It can be used to identify highly synergistic gene sets and to identify candidate biomarker genes or therapeutic targets based on the coherence of gene sets and the association between gene sets and phenotypes. Therefore, it is widely used in the association analysis between gene sets and disease or clinical features of patients and to identify candidate hub genes by calculating co-expression modules and analyzing gene and phenotypic correlations in modules [7,8,9,10]. The WGCNA is a systemic and powerful technique that uses gene expression data to construct a scale-free network, which requires a power operation on the correlation value and that defines as soft threshold. This power operation strengthens strong correlation, weakens weak correlation or negative correlation, makes the correlation value more in line with the characteristics of scale-free network, and gives the results biological significance. In present study, we described the key modules and hub genes with significant differences between nevus and SCM based on WGCNA and identified novel biomarkers associated with SCM prognosis through the prognosis association with Gene Expression Profiling Interactive Analysis (GEPIA) database.

## 2. Materials and Methods

### 2.1. Raw Data and Procession

We downloaded the count data for the human SCM dataset GSE98394 [11] from the Gene Expression Omnibus (GEO) database, which uses the Illumina HiSeq 2500 GPL16791 platform for high-throughput sequencing of 27 patients with common required nevus and 51 patients with primary melanoma. Of these, 44 patients with SCM had prognostic information.

First, we removed more than half of the genes that were not expressed in the sample of the expression profile and normalized the original data using log_2_ conversion and the preprocess Core package [12] in R 3.6.0. After background correction and quantile normalization, 18,673 genes were finally screened for WGCNA. The ENSG-ID of genes were converted to official gene symbols by the BioMart database (http://asia.ensembl.org/biomart/martview) [13].

### 2.2. Gene Co-Expression Network and Modules

We constructed a gene co-expression network through the WGCNA package [14]. First, we obtained the expression data and the phenotypic data matrix, removed the genes that were not expressed according to the expression profile, calculated the variance of each gene in each sample, screened the genes with the standard deviation >1.2, and further clustered all the samples. Some samples were far away, and outliers were excluded based on cluster distance. In order to filter outlier samples, the default value of −1 was applied, indicating that the program automatically removed outlier samples and selected the largest set of samples for subsequent analysis.

After constructing the scale-free network, the expression matrix was transformed into the adjacency matrix and converted into a topological matrix. Based on the topological overlap measure (TOM), we used the average-linkage hierarchical clustering method to cluster the genes and set the minimum base number of each gene network module to 30. When merging modules, a threshold for the combination of similar genes or modules needed to be set, which was called the module merge height threshold. The larger the threshold, the fewer modules were merged and the general value of this threshold could be 0.25. Therefore, after using the dynamic shear to identify the gene module, we calculated the eigengenes of each module one by one, then clustered the modules and merged the closer modules into new modules according to height = 0.25.

The correlation between modules and phenotypes was calculated according to the feature vector of each module. According to the expression level of each gene in each sample, the correlation between the genes in these modules and each phenotype was calculated to measure the gene significance (GS). The larger the value of the GS, the more biologically significant. In other words, GS = 0 indicates that the gene is not related to the phenotype.

### 2.3. Hub Gene Identification and Validation

Based on the eigenvectors of each module, we calculated the correlation of the expression of the genes in each module, and the genes with a correlation >0.9 were candidate hub genes. The expression levels of these genes were heat mapped using TBtool software [15].

Next, we divided the GSE98394 dataset into a nevus group and a melanoma group, and screened for the differentially expressed genes between the two groups by edgeR package [16,17]. The screening conditions were |log_2_FC| > 2, false discovery rate (FDR) < 0.05. The prognostic value genes were screened according to the prognostic information in this dataset. The patients were stratified into a high-level group and a low-level group according to median expression and the Kaplan–Meier method was used for survival analyses by GraphPad Prism version 8.0.0 for Windows. The log-rank test was used to compare the survival curves of patients in different subgroups and *P* < 0.05 was considered to indicate a statistically significant difference. Then, verification was performed by the GEPIA database (http://gepia.cancer-pku.cn) [18] to identify hub genes.

Finally, we analyzed the protein expression of hub genes in normal skin and in melanoma tissues by Human Protein Atlas (http://www.proteinatlas.org) [19]. The direct link to these images in the Human Protein Atlas are as follows: https://www.proteinatlas.org/ENSG00000134602-STK26/tissue/skin#img (STK26 in skin); https://www.proteinatlas.org/ENSG00000134602-STK26/ Pathology/tissue/melanoma#img (STK26 in melanoma); https://www.proteinatlas.org/ENSG00000162687-KCNT2/tissue/skin#img (KCNT2 in skin); https://www.proteinatlas.org/ENSG00000162687-KCNT2/pathology/tissue/melanoma#img (KCNT2 in melanoma). 

Immunohistochemical images were measured by ImageJ software [20] and analyzed by GraphPad Prism version 8.0.0 for Windows (San Diego, CA, USA, www.graphpad.com). Difference analysis between the two groups was performed by Student’s *t* test and *P* < 0.05 was considered statistically significant.

## 3. Results

### 3.1. Gene Expression Data

**Figure 1:**
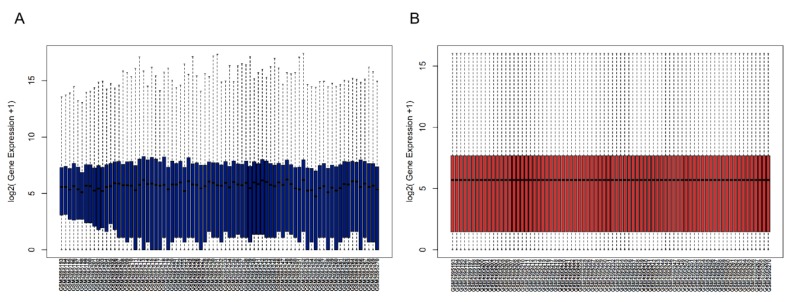
Box plots of gene expression data of GSE98394 datasets (**A**) before and (**B**) after normalization. The x-axis represents the gene expression level and the y-axis represents the samples.

After data processing, the expression profile data and phenotypic data matrix were obtained from the GSE98394 dataset, which contained 78 samples, 18,673 genes, and 7 phenotypes. The before and after normalizations are shown in Figure 1. As the figure shows, the average RNA expression of each sample was consistent after normalization and could be used for subsequent analysis.

### 3.2. Clinically Significant Modules

**Figure 2:**
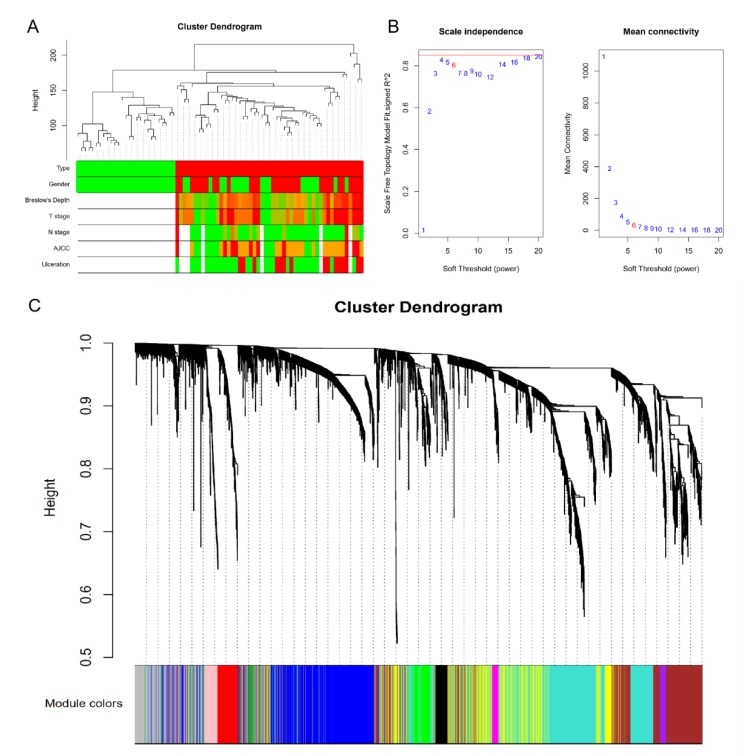
Gene co-expression network and modules. (**A**) Clustering dendrogram of 78 samples. (**B**) Determination of soft-thresholding power in the weighted gene co-expression network analysis (WGCNA). When β = 6, R^2^ > 0.8 and mean connectivity < 100, indicating that the network has the characteristics of scale-free topology. (**C**) Dendrogram of all differentially expressed genes clustered based on a dissimilarity measure. The cluster analysis result is shown above, and module identification is shown below.

After cluster analysis, a new data expression profile was obtained from the GSE98394 dataset, which contained 74 samples and 4863 genes. The clustering results with sample characteristics are shown in Figure 2A, where the red color indicates the samples marked as non-zero in the phenotypes.

**Figure 3:**
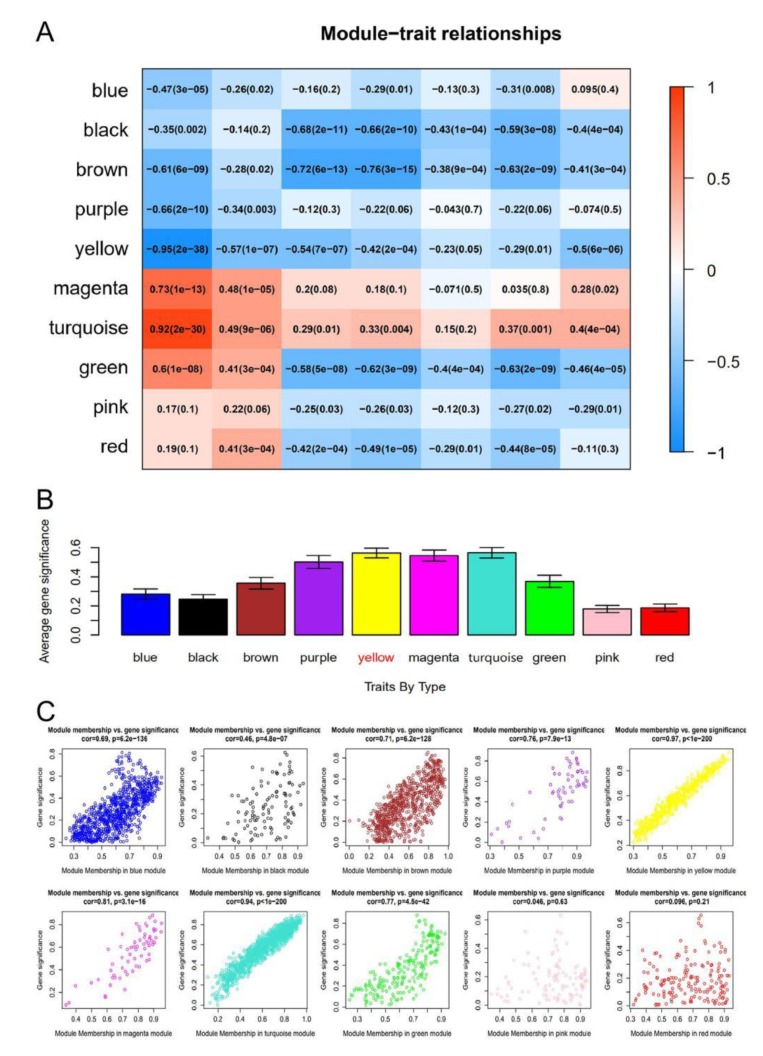
Identification of modules associated with tissue types. (**A**) Heatmap of the correlation between module eigengenes and clinical traits. Red means positive correlation, blue means negative correlation, and the darker the color, the close the absolute value is to 1, and the stronger the correlation. (**B**) Distribution of average gene significance and errors in the modules associated with tissue type. The x‑axis indicates the module, the y‑axis indicates the significance of overrepresentation. (**C**) Scatter plots of the degree and *P*-value of Cox regression in dataset. The x-axis indicates the degree of regression, the y-axis indicates the gene significant. Each circle represents a gene.

In this study, in order to ensure that the network was a scale-free network, we chose a soft threshold of β = 6. As shown in Figure 2B, R^2^ > 0.8 and mean connectivity <100 was reached after this power operation, indicating that the network has the characteristics of scale-free topology. A total of 11 modules were identified via the average linkage hierarchical clustering, as shown in Figure 2C. The yellow module was found to have the highest association with tissue types, as shown in Figure 3A–C.

### 3.3. Hub Genes 

**Figure 4:**
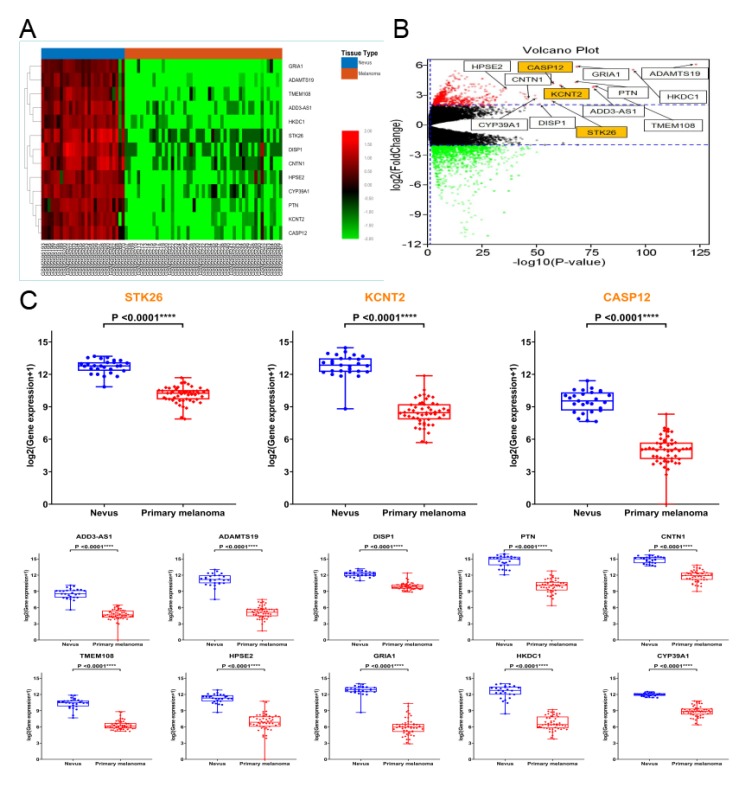
Candidate hub genes. (**A**) The heatmap of 13 candidate hub genes. The 13 genes were all upregulated in nevus compared to melanoma. (**B**) The volcano plot of 1276 differentially expressed genes. The 13 candidate hub genes are highlighted, and all were differentially expressed genes. (**C**) Gene expression levels of the 13 candidate hub genes between nevus and primary melanoma based on the GSE98394 datasets. Student’s *t* test was used to evaluate the statistical significance of differences.

By calculating the expression correlation of genes in the yellow module, those with a correlation >0.9 were considered candidate hub genes (Table 1). We heat mapped the expression of the 13 candidate hub genes, which showed that the 13 genes were all upregulated in nevus compared to melanoma (Figure 4A).

After screening by the edgeR package, 1276 differentially expressed genes were obtained (Figure 4B), and the 13 candidate genes were found to be differentially expressed genes. The mRNA levels of these 13 candidate genes showed statistically significant differences between nevus and primary melanoma based on the GSE98394 datasets (Figure 4C).

**Figure 5:**
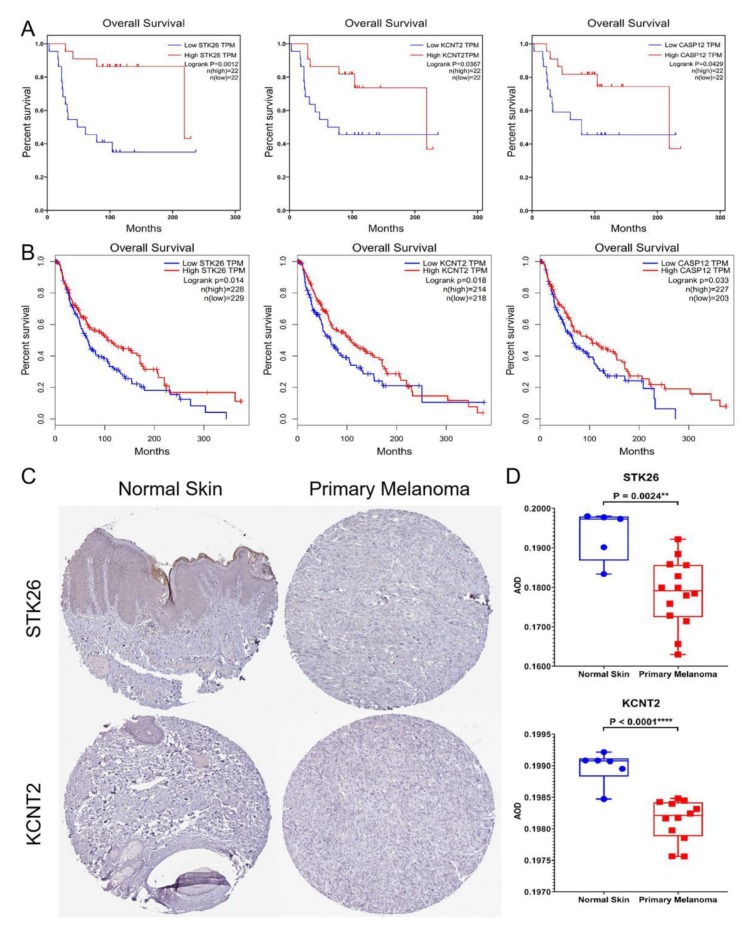
Hub gene identification and validation. (**A**) Overall survival of the three hub genes in skin cutaneous melanoma based on the GSE98394 datasets. The patients were stratified into high-level group and low-level group according to median expression. (**B**) To identify hub genes by the GEPIA database. The patients were stratified into high-level group and low-level group according to median expression. (**C**) Immunohistochemistry of STK26 and KCNT2 based on the Human Protein Atlas. (**D**) Gray-scale analysis revealed statistically significant differences in the protein expression of STK26 (*P* = 0.0024) and KCNT2 (*P* < 0.0001) between normal skin and melanoma.

We then analyzed the overall survival of patients in the GSE98394 datasets and stratified them into high-level and low-level groups according to the median expression of these 13 candidate genes for screening prognostic genes, and found that the low expression of the three genes *STK26*, *KCNT2*, and *CASP12* was associated with poor prognosis of patients with primary melanoma (Figure 5A). In addition, we verified this using the GEPIA database (Figure 5B). Therefore, these three genes were considered to be hub genes.

Based on the Human Protein Atlas database, we examined the expression of these three hub genes in normal tissues and primary melanoma and found that STK26 and KCNT2 had immunohistochemistry (IHC) images of normal tissues and melanoma tissues. We selected representative images for display (Figure 5C). In addition, gray-scale analysis revealed statistically significant differences in the protein expression of *STK26* (*P* = 0.0024) and *KCNT2* (*P* < 0.0001) between normal skin and melanoma (Figure 5D).

## 4. Discussion

Before targeted immunotherapy for SCM, advanced patients have limited treatment and poor prognosis, with a median survival of only 6–9 months and a 1-year survival rate of only 25% [21]. Since 2011, with the continuous approval of immunotherapy and targeted therapeutic drugs, the treatment of advanced melanoma has made a breakthrough. Similar to all solid malignancies, the prognosis of SCM depends on the stage of the visit. Usually, patients with localized disease and thickness of primary tumor ≤1.0 mm have a good prognosis, with a 5-year survival rate of over 90% [22]. Therefore, early diagnosis and early screening of SCM is quite necessary.

The development of diagnostic or prognostic gene detection techniques for SCM play a certain role in predicting the biological behavior of unknown histopathological features of atypical melanocyte-like lesions (such as atypical melanocyte hyperplasia, Spitz tumors with unclear malignant potential, etc.) [23]. There is a huge clinical demand for this technology, but the development of a true differential detection technology still faces challenges. Therefore, it is very important to explore specific biomarkers for distinguishing SCM from nevus. In this study, we used the gene expression dataset GSE98394 in the GEO database to screen for potential biomarkers associated with this process. The prognostic value of these biomarkers was verified by the prognostic information in this dataset and the GEPIA database, which was integrated with the TCGA and GTEx data.

WGCNA is an algorithm for searching module information from chip data. In this method, a module is defined as a group of genes with similar expression profiles. If some genes always have similar expression changes in a physiological process or in different tissues, these functionally related genes can be defined as a module. Candidate hub genes or therapeutic targets are identified based on the connectivity of the module and the association between the module and the phenotype. Compared to genes that only focus on differential expression, WGCNA uses thousands or nearly 10,000 of the most variable genes or all of the genes to identify modules of interest and conduct significant association analyses with phenotypes. One of the advantages is to make full use of information, and the other is to convert thousands of genes and phenotypes into several modules and phenotypes, eliminating the need for multiple hypothesis testing [7,8,24,25].

WGCNA was performed to explore gene co-expression modules associated with nevus and primary melanoma. A total of 18,673 genes and 7 phenotypes were used to constructed by a co-expression network and 11 modules were obtained. The yellow module was associated with the tissue type. In this module, there were 13 genes with a correlation >0.9. Among them, the low expression of the three genes (*STK26*, *KCNT2*, and *CASP12*) was associated with poor prognosis in patients with SCM.

*STK26*, serine/threonine kinase 26, also known as mammalian STE20-like protein kinase 4 (*MST4*), is a protein-coding gene. Early in vitro experiments showed that STK26 had biological effects in activating the MEK/ERK pathway, mediating cell growth, transformation, and regulating apoptosis [26,27]. In addition, in association with STK24, STK26 negatively regulated Golgi reorientation in polarized cell migration upon RHO activation [28]. Subsequent studies showed that *STK26* was a cancer-promoting gene in prostate cancer, liver cancer, and glioblastoma. In prostate cancer, *STK26* expression was upregulated, and it might be a good indicator for identifying prostate cancer because *STK26* expression was not detected in patients with benign prostatic hyperplasia [29]. In hepatocellular carcinoma (HCC), the high expression of *STK26* was associated with large tumor size, microvascular invasion, intrahepatic metastasis, and TNM grading of patients with advanced HCC, which was independent prognostic factor of the overall survival rate (*P* = 0.004) and time to recurrence (*P* = 0.001) of patients after hepatectomy [30]. In addition, the inhibition of STK26 suppressed autophagy and the tumorigenicity of glioblastoma cells, while its therapeutic targeting enhanced the antitumor effects of radiotherapy [31]. In breast cancer, in vitro experiments showed that knocking out *STK26* led to an enhanced vascular invasion of breast cancer cells [32]. Therefore, *STK26* might play a role as a tumor suppressor gene in breast cancer. To the best of our knowledge, there were no reports of *STK26* in the melanoma. Our results indicated that STK26 was downregulated in SCM compared to nevus, and its low expression was associated with poor prognosis of primary melanoma. Further research is needed to uncover the mechanism and role of STK26 in the transformation process of nevus into SCM. 

Potassium channels regulate excitability, epithelial ion transport, proliferation, and apoptosis [33]. Diseases associated with potassium sodium-activated channel subfamily T member 2 (KCNT2) include early infantile epileptic encephalopathies [34]. To the best of our knowledge, there are few studies on *KCNT2* in cancer research. Gunnarsson et al. [35] found that seven pediatric B-cell precursor acute lymphoblastic leukemia with dup (1q) revealed non-synonymous somatic single nucleotide variants in *KCNT2* by sequencing the breakpoint regions and all exons on 1q. There were no reports related to *KCNT2* in melanoma. The expression of *KCNT2* in melanoma was more downregulated than that of normal tissues at both the transcriptional and the protein level. Therefore, the expression of *KCNT2* may be an indicator of the identification of nevus and melanoma, but further research and large-scale clinical data should be carried out to confirm these hypotheses.

Caspases are cysteine proteases, which play a very important role in the regulation of inflammatory response and apoptosis. Among them, CASP12, also known as caspase-12, is usually classified as inflammatory caspase, which inhibits the activation of caspase-1, the production of pro-inflammatory cytokines IL-1b and IL-18, and the overall response to sepsis in inflammatory complexes, which results in negative regulation in inflammatory response [36]. Most studies have also focused on infection-related diseases and seldom on cancer. In vitro experiments showed that Casp12-deficient mice were more sensitive to drug-induced colon cancer [37]. Similarly, the Casp12 degraded IκBα protein and enhanced MMP-9 expression in human nasopharyngeal carcinoma (hNC) cell invasion [38]. Interestingly, it was found that knockdown of *CASP12* diminished *trans*-resveratrol-mediated apoptosis in hNC cells [39]. Our results showed that, similarly to *STK26* and *KCNT2*, the transcription of *CASP12* in melanoma was reduced relative to nevus, which might mediate the process of the conversion of nevus to melanoma. Unfortunately, there were no IHC results of Casp12 in the Human Protein Atlas database, so we could not analyze the difference in the protein expressions of *CASP12* between nevus and melanoma, which is worth further investigation.

## 5. Conclusions

In cancer research, co-expression analysis, as a powerful technical tool for analyzing multigenes and large-scale datasets, has been used in the analysis of a variety of cancers. In this work, we used WGCNA to screen out one module and three prognostic genes with a strong correlation in the difference between common nevus and SCM. Through the literature review, it was found that these three genes were not reported in the melanoma, although they were all related to apoptosis. Apoptosis is the basis of immunotherapy, which is an effective treatment for melanoma. Therefore, these three genes have further research value. In addition, after verification by the GEPIA database and IHC data from the Human Protein Atlas database, it was indicated that these three genes may have potential value as novel biomarkers in the early diagnosis and prognosis of SCM.

## Figures and Tables

**Table 1:** Candidate hub genes in the yellow module. Genes with a correlation (R) > 0.9 were candidate hub genes.

Genes Symbol	Full Name	R	*P*-Value
ADAMTS19	Disintegrin and Metalloprotease Domain (ADAM) Metallopeptidase with Thrombospondin Type 1 Motif 19	0.96	6.68 × 10^−40^
KCNT2	Potassium Sodium-Activated Channel Subfamily T Member 2	0.95	1.50 × 10^−39^
CASP12	Caspase 12	0.94	4.48 × 10^−39^
ADD3-AS1	Adducin 3 Antisense RNA 1	0.93	2.78 × 10^−33^
DISP1	Dispatched RND Transporter Family Member 1	0.93	1.57 × 10^−32^
PTN	Pleiotrophin	0.92	8.81 × 10^−32^
CNTN1	Contactin 1	0.92	1.94 × 10^−31^
TMEM108	Transmembrane Protein 108	0.92	1.61 × 10^−30^
HPSE2	Heparanase 2	0.92	2.14 × 10^−30^
GRIA1	Glutamate Ionotropic Receptor AMPA Type Subunit 1	0.91	6.86 × 10^−30^
HKDC1	Hexokinase Domain Containing 1	0.91	1.58 × 10^−29^
STK26	Serine/Threonine Kinase 26	0.91	1.36 × 10^−28^
CYP39A1	Cytochrome P450 Family 39 Subfamily A Member 1	0.90	3.26 × 10^−28^
